# Hypoxia-induced tRF-3^Thr-CGT^ promotes hepatocellular carcinoma progression via mitochondrial energy metabolism remodeling dependent on the mtDNA-translation mechanism

**DOI:** 10.3389/fphar.2025.1549373

**Published:** 2025-05-30

**Authors:** Xianzhi Qu, Buhan Liu, Duo Jin, Yue Ma, Mingjun Liu, Xiaoyu Yan, Jing Su, Lei Zhou

**Affiliations:** ^1^ Department of Hepatobiliary and Pancreatic Surgery, Second Hospital of Jilin University, Changchun, China; ^2^ Department of Pathology, Affiliated Hospital of Changchun University of Chinese Medicine, Changchun, China; ^3^ Key Laboratory of Pathobiology, Department of Pathophysiology, Ministry of Education, College of Basic Medical Sciences, Jilin University, Changchun, China; ^4^ Department of Clinical Laboratory, The Affiliated Hospital to Changchun University of Chinese Medicine, Changchun, China

**Keywords:** hypoxia, tRNA-derived fragment, hepatocellular carcinoma, mitochondrial, oxidative phosphorylation, energy metabolism remodeling

## Abstract

Hypoxia is one of the major characteristics of the tumor microenvironment, and it promotes mitochondrial energy metabolic remodeling for hepatocellular carcinoma (HCC) progression. It is believed that under dual control of the mitochondrial genome (mtDNA) and the nuclear genome (nDNA) mitochondria coordinate multiple signals to alter energy metabolism under hypoxic stress. Currently, it has been found that hypoxia promotes tRNA cleavage to produce tRFs (tRNA-derived fragment), which have attracted attention as potential biomarkers and therapeutic targets. In this study, we found that hypoxic stress could drive HCC cell invasion and migration. Furthermore, the expression of core oxidative phosphorylation (OXPHOS) proteins encoded by nDNA and mtDNA were uncoordinated under hypoxia. Therefore, the human mitochondrial peptide deformylase (HsPDF) which was essential for mtDNA-encoded protein translation and respiratory chain maintenance has been brought into focus. We found that hypoxic stress significantly suppressed HsPDF which was responsible for mtDNA-encoded protein inhibition. To further explore the possible mechanism, high-throughput sequencing was used to map tRF expression patterns in HCC cells under hypoxia. We found that hypoxic stress altered their subtype distributions and that the high expression of tRF-3^Thr-CGT^, which has functions in transcription and translation regulation, may potentially bind to the 3ʹ-UTR of HsPDF. Upregulated tRF-3^Thr-CGT^ could inhibit HsPDF and mitochondrial OXPHOS function. Furthermore, the orthotopic liver cancer model in mice also indicated that the tRF-3^Thr-CGT^ inhibitor significantly suppressed tumor progression. These results collectively suggested that tRFs may have roles in mitochondrial protein coordination and become novel pharmacological targets for mitochondrial remodeling under tumor microenvironment remodeling of HCC therapy.

## 1 Introduction

Hepatocellular carcinoma (HCC) is one of the deadliest malignant tumors worldwide (Llovet et al., 2021; Siegel et al., 2024). Despite recent breakthroughs in surgery, chemotherapy, and transarterial chemoembolization for advanced HCC treatment, recurrence caused by high metastasis rates have become major reasons for poor prognosis in patients with HCC (Galle et al., 2021; Cai et al., 2022). It is suggested that because of dysfunctional angiogenesis, hypoxia plays central roles in regulating the metabolic reprogramming of HCC (Yang et al., 2020; Zhong et al., 2022). To our knowledge, under dual control of the mitochondrial (mtDNA) and the nuclear genomes (nDNA), mitochondria function as key oxygen sensors and may frequently coordinate multiple signals to alter energy metabolism for HCC metastasis and survival (Bao et al., 2019; Zhang et al., 2022). However, fundamental gaps remain in our understanding of the crosstalk between mitochondria and the nucleus which limit further improvement of therapeutic strategies.

As high-throughput technology developed new types of non-coding RNA, tRNA-derived fragments (tRFs and tiRNAs) have been explored (Fu et al., 2023a). Under certain types of pressure stimulation, they may be formed by selective tRNA to regulate the expression of oncotarget genes and participate in important pathways related to tumor metastasis (Salehi et al., 2024). tiRNAs are generated from halves of tRNA, while tRFs mainly contain tRF-1, tRF-2, tRF-3, and tRF-5 according the cleavage location (Yang et al., 2023). Current studies indicate that energy stress and reactive oxygen species (ROS) brought about by hypoxia promote RNase to cleave tRNA at T-loop, D-loop, and anti-codon regions for tRF formation which regulated protein translation, gene silencing, and RNA processing (Li et al., 2021). Skeparnias et al. found that upregulated tRF-5003b affected genes involved in networks that regulate the mTOR signaling pathway, transcription and translation regulation, as well as the structural constituents of ribosomes (Skeparnias et al., 2020). Furthermore, tRFs are also enriched in saliva from patients with tumors and their expression is closely related to prognosis. Due to their high accuracy and tissue specificity, tRFs have potential as tumor biomarkers (Li et al., 2022)].

It is known that mtDNA encodes 13 proteins that are essential for the respiratory complex and oxidative phosphorylation (Yoshinaga and Numata, 2022). In contrast to the translation of nuclear genome-encoded proteins, the formyl group of N-terminally formylated peptides derived from the proteins encoded by mtDNA are selectively removed by human mitochondrial peptide deformylase (HsPDF) (Escobar-Alvarez et al., 2010). Inhibiting HsPDF could significantly disrupt mtDNA-related translation, specifically in cancer cells (Sheth et al., 2014; Escobar-Alvarez et al., 2010; Fuhrmann and Brüne, 2017; Liu et al., 2022) it has been found that, under hypoxic stress, respiratory chain complex components may possess adaptive recombination for suppressing mitochondrial oxidative phosphorylation (OXPHOS), which is a critical factor for invasion and migration promotion. As tRNA shuttles through the nucleus and cytoplasm at a quantity that is 60–100 times that of mRNA, understanding whether tRFs participate in mtDNA-encoded proteins may provide new understanding of the crosstalk between mitochondrial and nuclear signaling for HCC survival and metastasis under hypoxic conditions.

In this study, we focus on mitochondrial energy metabolic remodeling caused by mtDNA-encoded protein translation suppression during hypoxia, which may be critical for HCC cell Invasion and migration. Using RNA sequencing, we investigated the roles of hypoxia-induced tRF-3^Thr-CGT^ in regulating mtDNA-encoded protein translation through manipulating HsPDF expression. We aimed to clarify whether tRFs induced by hypoxic stress may function as messengers for the crosstalk between mitochondria and the nucleus and provide new strategies for anti-HCC therapy.

## 2 Materials and methods

### 2.1 Cell culture

The human hepatocellular carcinoma cell line SNU-449 (grade II-III/IV), SNU-387 (grade IV/V) and mouse liver cancer cell line Hepa1-6-LUC were cultured in Dulbecco’s modified Eagle’s medium (Gibco Life Technologies, Carlsbad, CA, United States) supplemented with 10% fetal bovine serum (Invitrogen, Carlsbad, CA, United States).

### 2.2 Scratch Healing Assay

SNU-387 and SNU-449 cells were seeded in 12-well plates, and wounds were made using a 10 µL pipette tip at time 0 h. After washing with PBS, SNU-387 cells were incubated with a medium supplemented with 2% fetal bovine serum, and SNU-449 cells were incubated with a serum-free medium. The cells were placed in a 37°C, 5% CO_2_ incubator for culture. Images were captured at 0, 24, and 48 h.

### 2.3 Migration assay

Migration assays were conducted using transwell chambers (Corning Incorporated, Kennebunk, ME, United States). Cells were suspended in a serum-free medium and seeded into the upper compartment of the chamber, and the lower compartment was filled with a medium supplemented with 20% fetal bovine serum. The chamber was incubated for 48 h at 37°C in 5% CO_2_. Cells on the upper surface of the membranes were removed by wiping with a cotton swab. The cells were stained with crystal violet for 15 min and mages were acquired by an Echo-lab Revolve microscope (ECHO, San Diego, CA, United States).

### 2.4 tRFs and tiRNA sequencing

tRF and tiRNA are heavily decorated by RNA modifications that interfere with small RNA-seq library construction.RNA samples: 3′-aminoacyl (charged) deacylation to 3′-OH for 3′adaptor ligation, 3′-cP (2′,3′-cyclic phosphate) removal to 3′-OH for 3′adaptor ligation, 5′-OH (hydroxyl group) phosphorylation to 5′-P for 5′-adaptor ligation, m1A and m3C demethylation for efficient reverse transcription. Sequencing libraries are size-selected for the RNA biotypes to be sequenced using an automated gel cutter. The libraries are qualified and absolutely quantified using Agilent BioAnalyzer 2100. For standard small RNA sequencing on Illumina NextSeq instrument, the sequencing type is 50 bp single-read. Diluted libraries were loaded onto reagent cartridge and forwarded to sequencing run on Illumina NextSeq 500 system using NextSeq 500/550 V2 kit (#FC-404-2005, Illumina), according to the manufacturer’s instructions.

### 2.5 RNA extraction and reverse transcription-quantitative PCR (RT-qPCR) analysis

Total RNA was extracted from tissues or cells using TRIzol^®^ reagent (Invitrogen; Thermo Fisher Scientific, Inc.) and reverse-transcribed into cDNA according to the manufacturer’s instructions, by using the EasyScript First-Strand cDNA Synthesis (Beijing Transgen Biotech Co., Ltd.). Quantitative real time PCR was done by using the MX300P instrument (Agilent, United States) followed by a 3-step PCR protocol. The primer se-quences were as follows: RT-stemloop primer for tRF-3^Thr-CGT^: 5′-GTC​GTA​ACC​CCG​TCC​GTG​C-3′. Forward primer for tRF-3^Thr-CGT^: 5′-ACT​GTT​GAA​TTT​CCT​GGA​TGT​GTC-3′. Universal q-PCR reverse primer for ^tRF-3Thr-CGT^: 5′-CCA​GTG​CAG​GGT​CCG​AGG​TA-3′. Primers for ATP6: 5′-CCA​TGG​CCA​TCC​CCT​TAT​GA-3′, 5′-TGTTGTCGTGCAGGT AGAGG-3′. Primers for mtND1 5′-TGC​TTA​CCG​AAC​GAA​AAA​T-3′, 5′-GAG​TTG​GTC​GTA​GCG​GAA-3′. Primers for CYTB: 5′-GAAGGGCAAGATGAA GTGAAAG-3′, 5′-TTA​CTA​TCC​GCC​ATC​CCA​TAC-3′. Primers for COX2: 5′-TCA​AGT​CCC​TGA​GCA​TCT​ACG​GTT-3′, 5′- CTG​TTG​TGT​TCC​CGC​AGC​CAG​A TT-3′. Primers for Actin: 5′-AGC​AGC​ATC​GCC​CCA​AAG​TT-3′, 5′-GGGCACGAAG GCTCATCATT-3′. Transfections were performed using Lipofectamine 3000 (Invitrogen, Carlsbad, CA, United States). The negative control (NC) group means that the cells transfected with the unrelated oligonucleotides.

### 2.6 Western blot

SNU-387 and SNU-449 cells were lysed with a protein extraction reagent buffer containing a protease inhibitor cocktail and phenylmethylsufonyl fluoride. Proteins (70 µg) were separated by 10% SDS-PAGE and transferred to a PVDF membrane. The membrane was blocked with 10% milk and washed with TBS + 0.1% Tween-20. Subsequently, the membrane was incubated with a primary antibody, anti-OXPHOS (Invitrogen #45-8199), anti-ATP6 (Proteintech#24169-1-AP), anti-CYTB (Proteintech#55090-1-AP), anti-HsPDF (Proteintech#24842-1-AP) and anti-actin (Proteintech# 66009-1-Ig) diluted 1:1,000 overnight at 4°C, followed by incubation with the secondary antibody. Immunodetection was performed using ECL reagent (Thermo Fisher Scientific, Inc.) and visualized by Syngene Bio Imaging (Synoptics Ltd.). ImageJ (×64) software (National Institutes of health) was used to quantify the results.

### 2.7 Oxygen consumption rate (OCR)

Cells were seeded in 384-well plates at a density of 5 × 10^3^ cells per well. After 24 h, the cells were dissolved in fresh complete DMEM for 3 h. The medium was then replaced with 80 μL of reaction mixture (5 μL of reconstituted MitoXpress^®^ Xtra reagent [Luxcel Biosciences Cork] and 75 μL of DMEM with actinonin and/or TMZ) per well. HS Mineral Oil was added to each well as a blocking agent and the plate was analyzed by CLARIOstar microplate reader (BMG Labtech).

### 2.8 Luciferase reporter assay

Luciferase reporter vectors of Wild-type and mutant HsPDF (Site1:1168-1185 and Site2: 3447-3464) targeting the tRF-3^Thr-CGT^ binding site were constructed. Cells were co-transfected with the vectors and tRF-3^Thr-CGT^ mimics via Lipofectamine 3000. This assay was performed to assess luciferase activity 24 h after transfection according to the manufacturer’s protocol (Promega, Madison, WI, United States).

### 2.9 *In vivo* xenograft experiments

SPF grade C57BL/6J mice (male, 4–5 weeks old, 15–20 g body weight) were obtained from Beijing Viton Lever Laboratory Animal Co. They were housed in an SPF (Specific Pathogen-Free) grade laboratory facility with a 12-h light/dark cycle and maintained at a temperature of 22°C ± 2°C with 50%–60% humidity. Mice were randomly assigned to three groups. Synthetic analogs, inhibitors of tRF-3^Thr-CGT^ and negative unrelated oligonucleotides (7 μg each) were transfected into Hepa1-6-LUC cells at the logarithmic growth phase (5 × 10^6^ cells/well) using Lipofectamine 3000 (Invitrogen, Carlsbad, CA, United States). The cells were then digested, centrifuged, and resuspended in a mixture of PBS and Ceturegel^®^ High Concentration Matrix Gel (1:1, v/v) at a volume of 0.1 mL. Mice were anesthetized with 2.5% averantin, secured in a supine position, and a 1-cm midline incision was made along the linea alba. The transfected cell suspension with matrix was injected into the left liver of mice. The incision was closed with 5-0 silk sutures and disinfected with iodine. *In situ* hepatocellular carcinoma was imaged *in vivo* 7 days post-surgery. Mice were monitored daily for general health, behavior and any signs of distress. When shown 20% loss in body weight or severe distress animals were humanely euthanized in accordance with our ethical guidelines. All animal experiments were performed according to the National Guidelines for Experimental Animal Welfare, with approval from the Animal Welfare and Research Ethics Committee at Jilin University (Changchun, China) [Approval Number. 2020034]. Laboratory animal use license number (SYXK (Ji)2018-0001).

### 2.10 Optical *in vivo* imaging

Live imaging system for small animals was used to investigate tumor growth. Anesthetize the mice with 2.5% afodin before imaging. When the mice are breathing steadily, injected 10 μg/mL of substrate D-luciferin 200 µL intraperitoneally and perform fluorescence *in vivo* imaging 10 min after substrate injection. Place the mouse into the platform of imaging system. Measurements and data processing and image preservation work are carried out on areas of the mouse liver.

### 2.11 H&E staining

Tissue samples were fixed and processed through 10% neutral buffered formalin and embedded in paraffin. Paraffin sections (4 µm) were dewaxed, rehydrated, and stained with hematoxylin for 7 min, followed by differentiation in 1% acid alcohol and counterstaining with eosin for 3 min. Representative images were captured using microscope for analysis.

### 2.12 Statistical analysis

All results were obtained from at least three independent experiments and are expressed as means ± SD (n = 3). Statistical analysis was carried out using GraphPad Prism 8.0. Statistical significance was calculated using t-test (unpaired/paired), a *p*-value <0.05 was considered statistically significant (*p < 0.05, **p < 0.01 and ***p < 0.001).

## 3 Results

### 3.1 Hypoxia promoted the invasion and migration ability of HCC cells

Studies found that lower oxygen concentration was associated with a higher tendency of metastasis in HCC (Wang et al., 2023). SNU-387 and SNU-449 cells were cultured under 1% O_2_, and 94% N_2_ conditions for 24 h as previous studies (Yan et al., 2021). Transwell and scratch experiments were used to detect invasion and migration ability. The results showed that compared with HCC cells under normoxia, hypoxia significantly promoted the invasion and migration abilities due to hypoxic stress stimulation (Supplementary Figures S1A–D).

### 3.2 Expression of proteins encoded by mtDNA were suppressed under hypoxia in HCC cells

**FIGURE 1:**
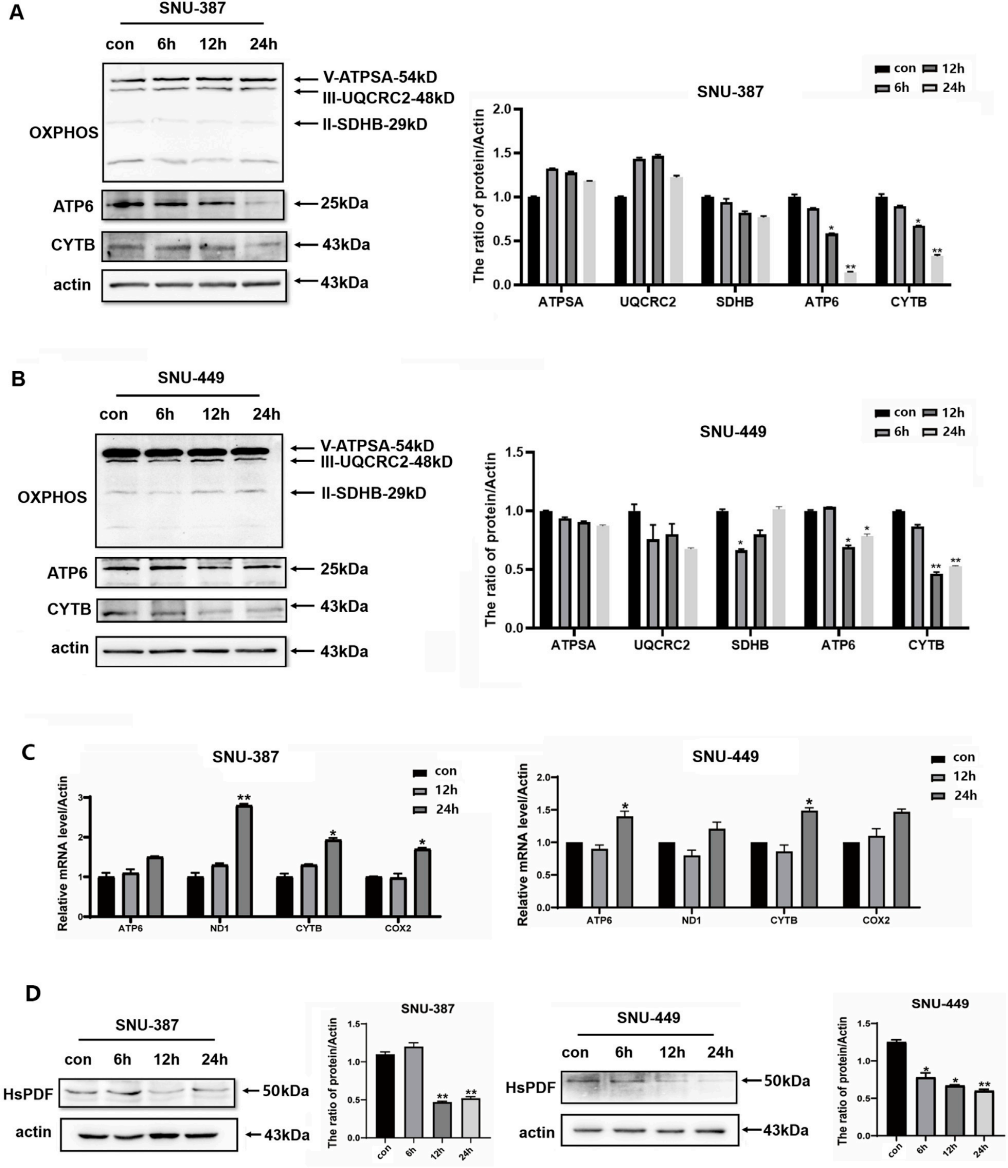
Hypoxia inhibited expression of mtDNA-encoded proteins and HsPDF in HCC cells. **(A,B)** The protein expression of ATPSA, UQCRC2, SDHB, ATP6 and CYTB in SNU-387 and SNU-449 cells were measured by Western blotting. **(C)** Relative ATP6, ND1, CYTB and COX2 expression in SNU-387 and SNU-449 cells was measured by qRT-PCR. **(D)** The protein expression of HsPDF in SNU-387 and SNU-449 cells was measured by Western blotting. Data are presented as mean ± SD, n = 3, *p < 0.05, **p < 0.01.

It is known that the OXPHOS system has components encoded by dual genome, while mtDNA-encoding proteins must rigorously match requirements to assemble functional respiratory complexes (Lechuga-Vieco et al., 2021). Thus, to investigate the change of OXPHOS complexes induced by hypoxia, Western blotting was used to detect the expression of typical mitochondrial proteins in SNU-387 and SNU-449 cells. The results showed that compared with complex subunits encoded by nDNA, such as ATPSA (ATP synthase), UQCRC2 (complex III), and SDHB (complex II), the expression of ATP6 (ATP synthase) and CYTB (complex III) encoded by mtDNA were significantly decreased (Figures 1A,B). After investigating mRNA expression, we found that ATP6 and CYTB remain increased at the transcription level under hypoxia. Furthermore, the mRNA expression of other mtDNA-encoded proteins such as ND1 and COX2 was also upregulated (Figure 1C). These findings bring protein translation mechanisms in the mitochondria into focus, necessitating further investigation.

### 3.3 HsPDF inhibition was responsible for mtDNA-encoded protein downregulation under hypoxia

**FIGURE 2:**
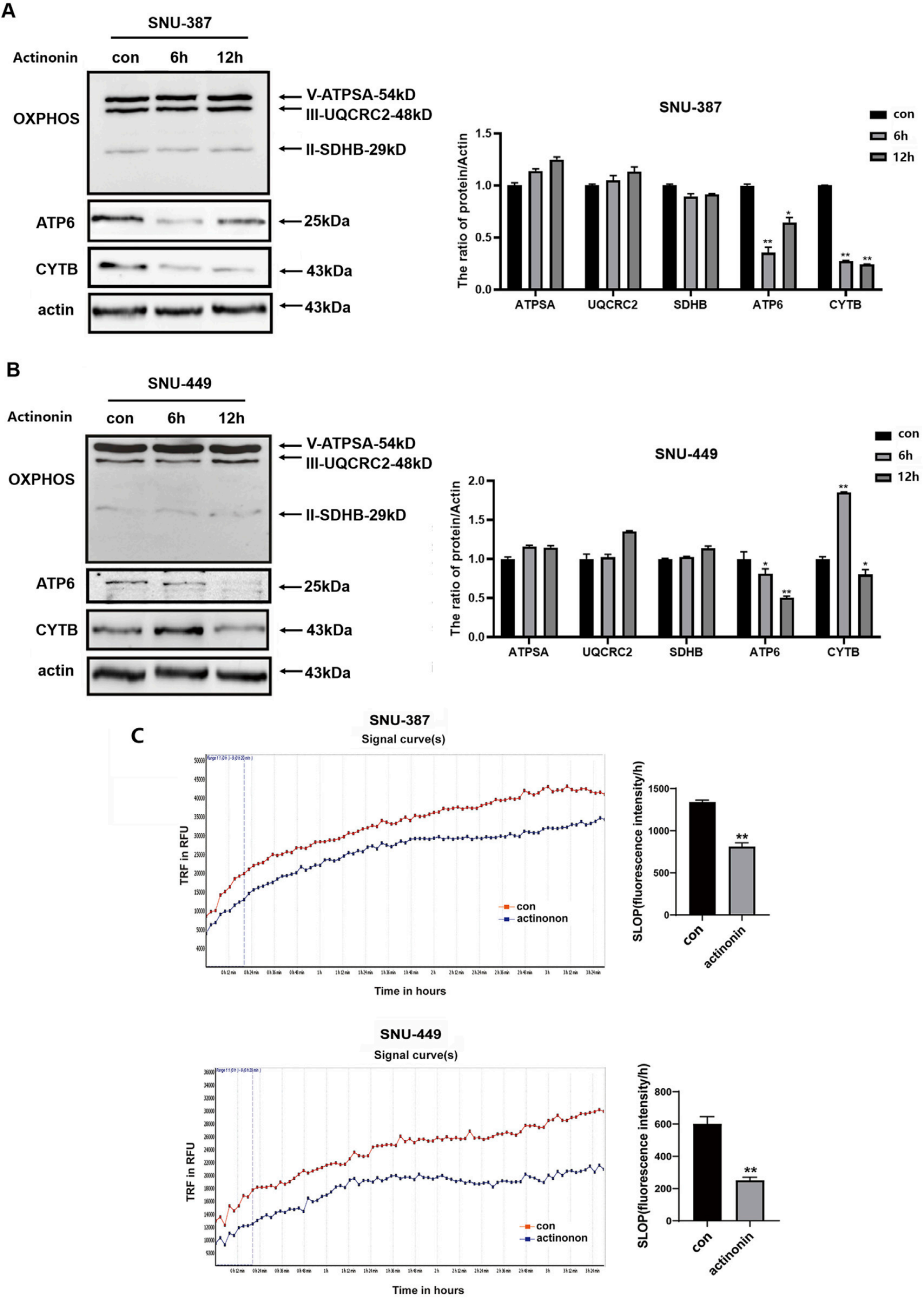
HsPDF inhibition was responsible for mtDNA encoded proteins downregulation and OXPHOS suppression under hypoxia. **(A,B)** HCC cells were treated with 40 μM actinonin and protein expression of HsPDF, ATPSA, UQCRC2, SDHB, ATP6 and CYTB were measured by Western blotting. **(C)** The oxygen consumption rate was quantified in SUN-387 and SNU-449 cells with actinonin treatment. Data are presented as mean ± SD, n = 3. *p < 0.05, **p < 0.01.

The peptide deformylase HsPDF is necessary for removing N-formyl-methionine from the newly synthesized peptides to achieve their proper folding, which differs from translation mechanism in the cytoplasm (Escobar-Alvarez et al., 2009). We explored the expression of HsPDF in SNU-387 and SNU-449 cells, and the results showed that HsPDF decreased significantly under hypoxia (Figure 1D). To further confirm whether HsPDF was responsible for mitochondrial protein translation dysfunction under hypoxia, the HsPDF inhibitor actinonin was used to explore its roles in HCC cells. The results showed HsPDF inhibition could selectively downregulate CYTB and ATP6 but ATPSA, UQCRC2, and SDHB expression (Figures 2A,B). Furthermore, HsPDF inhibition impaired the oxygen consumption rate in SNU-387 and SNU-449 cells (Figure 2C). These results indicated that HsPDF-mediated mitochondrial protein translation may be responsible for mitochondrial energy metabolism remodeling under hypoxia.

### 3.4 Hypoxic stress alters tRF expression patterns in HCC cells

**FIGURE 3:**
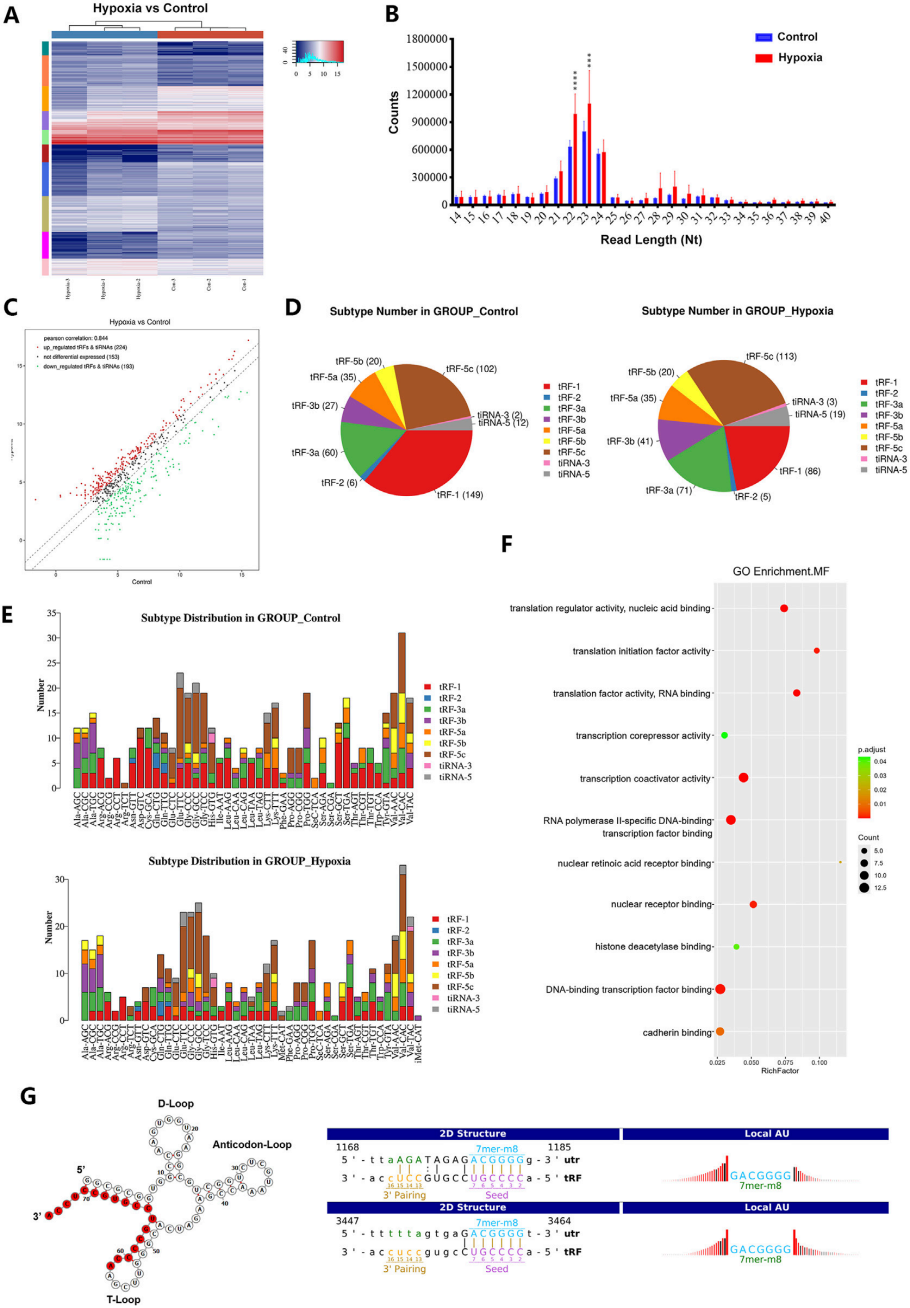
Hypoxic stress alters tRFs expression pattern in HCC cells. **(A)** The heatmap under normoxic (Control Group) and hypoxic condition (Hypoxia Group) of SNU-387 cells. Blue represents below-average expression levels and red represents above-average. **(B)** The tRFs reads length distribution between normoxia and hypoxia, hypoxia vs. normoxia group, Data are presented as mean ± SD, n = 3, ***p < 0.001 **(C)** Volcano plot of tRFs expression. Red/green indicates tRFs with statistically significant differences, with a fold change of no less than 1.5 and a p value ⩽0.05 (red: upregulated; green: downregulated). Gray indicates tRFs that are not statistically significant. **(D,E)** Subtype number and distribution in normoxic and hypoxic groups. **(F)** Metabolic pathways and signaling pathways enriched for tRF-3^Thr-CGT^ target genes according to GO analysis. **(G)** The structure of tRF-3^Thr-CGT^ and its sequences complementary to the 3′UTR of HsPDF (1168-1185, 3447-3464) with miRanda sequence alignment.

Hypoxia has been reported to induce tRFs and tiRNAs and become hotspot as novel post-transcriptional gene regulatory mechanisms. To explore their roles in hypoxia-induced HCC progression, we performed high-throughput sequencing on SNU-387 cells under hypoxic conditions. The heatmap and reads length distribution showed that hypoxia changed the overall expression and distribution of tRFs and tiRNAs (Figures 3A,B). Comparing subtype distributions between normal and hypoxia groups also proved that hypoxia altered tRF expression patterns in HCC cells (Figure 3E). Furthermore, hypoxia significantly upregulated 224 tRFs and tiRNAs while 193 were downregulated. Among them, tRF-3a (60/71) and tRF-3b (27/41) were the main upregulated types, which indicated that tRF-3 may function more key roles in hypoxia-induced HCC progression (Figures 3C,D). After conducting GO analyses to predict the potential mechanism of the highly expressed tRF-3 under hypoxia, we found the tRF-3a type tRF-58:75 Thr CGT-2M2 (hereinafter referred to as tRF-3^Thr-CGT^) was significantly related to the DNA-binding transcription factor and translation initiation factor activity (Figure 3F). Furthermore, miRanda sequence alignment suggested that there were two sequences complementary to the 3ʹ-UTR of HsPDF (1168-1185347-3464) (Figure 3G). These findings will aid in exploring tRF-3^Thr-CGT^ functions during hypoxia in HCC progression in more detail in future studies.

### 3.5 Hypoxia-induced tRF-3^Thr-CGT^ may target HsPDF and contribute to mitochondrial energy metabolism remodeling

**FIGURE 4:**
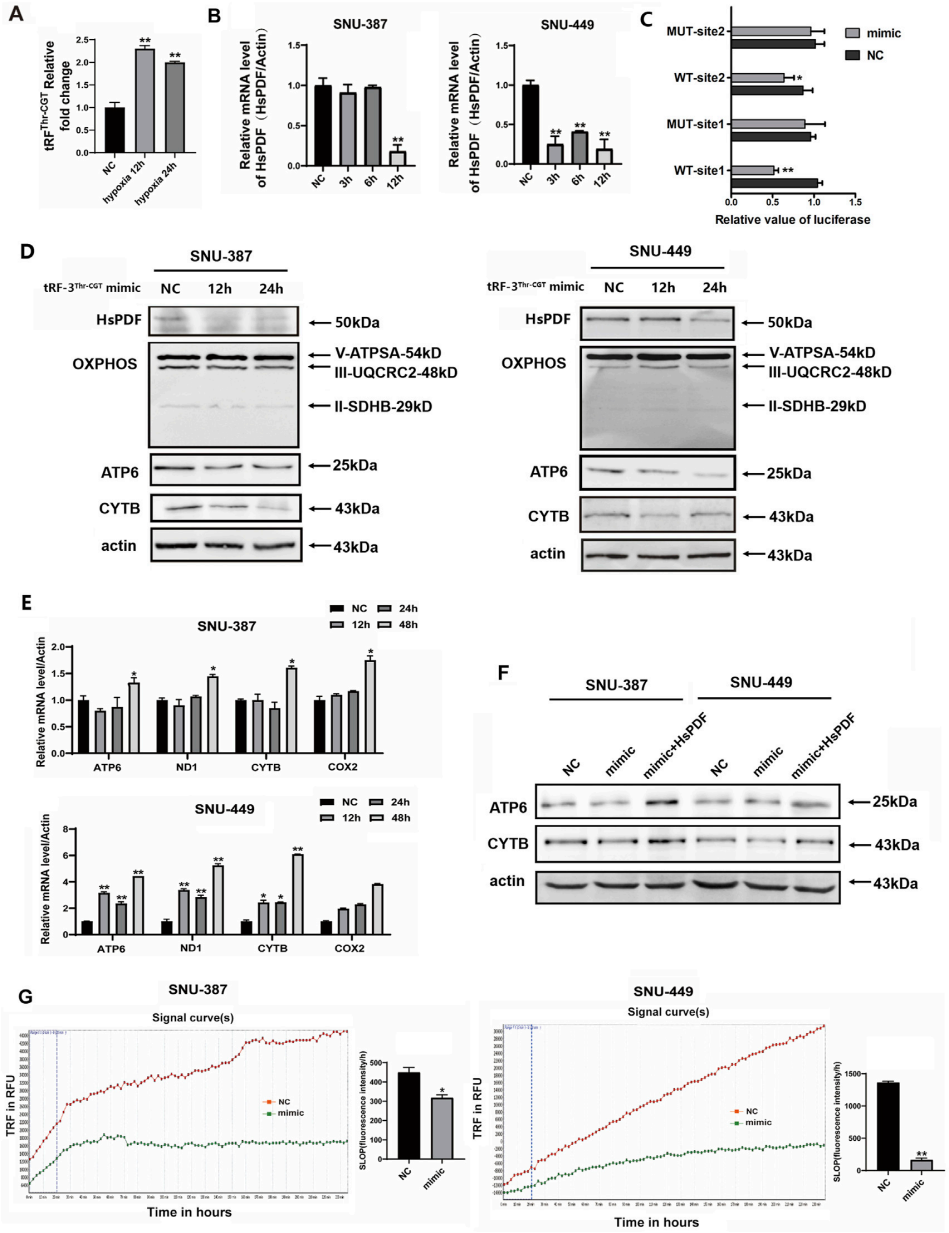
Hypoxia-induced tRF-3^Thr-CGT^ inhibited HsPDF and mitochondrial OXPHOS. **(A)** Relative tRF-3^Thr-CGT^ expression in SNU-387 cells was measured by qRT-PCR after 12 and 24 h hypoxia. **(B)** The mRNA expression of HsPDF with tRF-3^Thr-CGT^ mimic transfection in SNU-387 and SNU-449 cells was measured by qPCR. **(C)** The HsPDF WT or MUT reporter plasmid was co-transfected with NC and mimics into SNU-387 cells. Site1 was 1168-1185 and Site2 was 3447-3464 in 3′UTR of HsPDF. **(D)** The protein expression of HsPDF, ATPSA, UQCRC2, SDHB, ATP6 and CYTB were measured by Western blot in SNU-387 and SNU-449 cells with tRF-3T^hr-CGT^ mimic transfection. **(E)** The mRNA expression of ATP6, ND1, CYTB and COX2 with mimic transfection in SNU-387 and SNU-449 cells were measured by qPCR. **(F)** The protein expression of ATP6 and CYTB were measured in SNU-387 and SNU-449 cells overexpressed mimic and HsPDF. **(G)** The oxygen consumption rate was quantified in SUN-387 and SNU-449 cells with mimic transfection. Data are presented as mean ± SD, n = 3. *p < 0.05, **p < 0.01.

To explore the roles of tRF-3^Thr-CGT^ in HsPDF and mitochondrial function regulation of HCC, we first verified the expression of tRF-3^Thr-CGT^ under hypoxia using specific primers. Consistent with the high-throughput sequencing results, tRF-3^Thr-CGT^ was upregulated in SNU-387 cells (Figure 4A). We then transfected tRF-3^Thr-CGT^ mimic into hepatocellular carcinoma cells and the results showed that overexpressing mimic significantly inhibited HsPDF mRNA and protein expression (Figures 3B–D). According to the dual luciferase assay, mimic overexpression substantially inhibited wild-type HsPDF activity but not that of mutants, which supported tRF-3^Thr-CGT^ may regulated the 3′-UTR of HsPDF mRNA (Figure 4C). Similar with that under hypoxia, tRF-3^Thr-CGT^ mimic also suppressed mtDNA-encoding proteins expression while its influence on ATPSA, UQCRC2, and SDHB was limited (Figure 4D). We further confirmed the mRNA expression of mtDNA-encoded proteins and the results suggested tRF-3^Thr-CGT^ mimic also upregulated their expression as they were under hypoxic stimulation (Figure 4E). Meanwhile, rescue assays also showed that co-transfection of mimic and HsPDF could upregulated ATP6 and CYTB expression (Figure 4F). Finally, we explored how tRF-3^Thr-CGT^ mimic affected mitochondrial OXPHOS and found it decreased mitochondrial oxygen consumption in both types of HCC cells (Figure 4G).

### 3.6 Hypoxia-induced tRF-3^Thr-CGT^ promoted HCC cell invasion and migration

**FIGURE 5:**
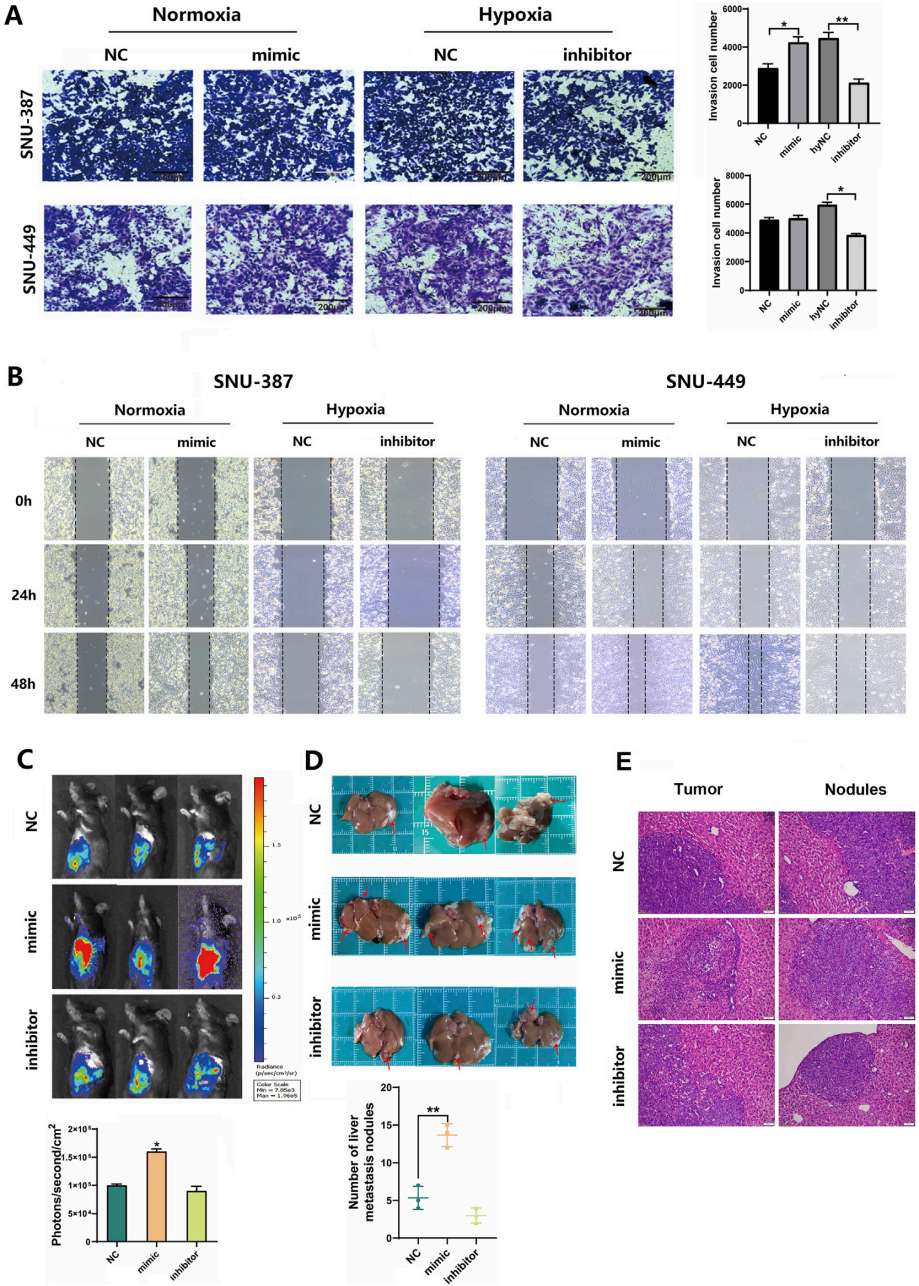
Hypoxia-induced tRF-3^Thr-CGT^ promoted HCC cells invasion and migration. **(A)** Transwell migration assays in SNU-387 and SNU-449 cells with tRF-3^Thr-CGT^ mimic transfection (bar = 200 μm). **(B)** Scratch Healing Assay at indicated time points in SNU-387 and SNU-449 cells. **(C,D)** Hepa1-6-LUC cells transfected with tRF-3^Thr-CGT^ mimic and inhibitor for orthotopic liver cancer model in mice. The fluorescence intensity and tumor nodules have been analyzed. **(E)** The representative images of H&E staining from tumor tissues and metastatic nodules. Scale bars = 50 μm. Data are presented as mean ± SD, n = 3. *p < 0.05, **p < 0.01.

To examine the effects of tRF-3^Thr-CGT^ on tumor invasion and migration, we transfected tRF-3^Thr-CGT^ mimic and inhibitor into SNU-387 and SNU-449 cells. The results showed that upregulated tRF-3^Thr-CGT^ under normoxia condition could promote cell invasion, especially migration ability. Conversely, tRF-3^Thr-CGT^ inhibition significantly limited cell invasion and migration under hypoxic stress (Figures 5A,B). Furthermore, the mouse *in situ* HCC model has been established to clarify the roles of tRF-3^Thr-CGT^ *in vivo*. We transfected tRF-3^Thr-CGT^ mimic and inhibitor into Hepa1-6-LUC cells and observed tumor nodule progress. The results showed that the fluorescence intensity of the NC group was 1.0 × 10^5^ ± 3,759 (ph/s/mm^2^), while that of the tRF-3^Thr-CGT^ mimic was 1.6 × 10^5^ ± 4,934 (ph/s/mm2). tRF-3^Thr-CGT^ inhibitor group was 7 × 10^4^ ± 8,334 (ph/s/mm^2^) (Figure 5C). Furthermore, the tRF-3^Thr-CGT^ inhibitor showed better retention in tumor nodules while the number of migration nodules were significantly increased in the tRF-3^Thr-CGT^ mimic group (Figure 5D). H&E staining also observed disordered arrangement of cells, with cellular atypia and indistinct cell nuclei in both tumor tissues and metastatic nodules (Figure 5E). These results suggested that hypoxia-induced tRF-3^Thr-CGT^ may become the potential target for HCC therapy.

## 4 Discussion

As current treatment for HCC to block angiogenesis provides limited benefit for patients, further understanding of the consequences caused by the tumor microenvironment such as hypoxia is necessary. Increasing evidence has revealed close relationships between non-coding RNAs and HCC progression (Fu et al., 2023b). With the development of high-throughput sequencing technology, tRF function has recently drawn wide attention. This study investigated how mitochondrial translation was affected by tRF signaling from tRNA in the cytoplasm and proposed models of translational plasticity that modulate mitochondrial translation in response to hypoxia. Furthermore, hypoxia-induced tRF-3^Thr-CGT^ was identified as a potential target for suppressing HCC invasion and migration.

Mitochondria are the main energy producers and oxygen consumers, implying that a hypoxic environment could severely affect OXPHOS. To adapt to decreased oxygen, mitochondria alter electron transport chain components, avoiding uncontrolled ROS production and contributing to HCC survival as well as metastasis (Mohammadalipour et al., 2020). Our study found that HCC cells stopped most mitochondrial OXPHOS function and showed increased migration and tendency for invasion under hypoxia. Recent evidence has shown that mitochondrial protein synthesis regulated by mitochondrial alanyl-tRNA synthetase 2 was impaired under hypoxia (Mao et al., 2024). Therefore, we investigated the effect of hypoxia on mitochondrial subunits and found the significant downregulated of mtDNA-related CYTB and ATP6 in protein levels, which brought the mitochondrial protein translation process into focus. HsPDF, which functioned as a specific enzyme for removing the formyl group of N-terminally formylated peptides derived from the proteins encoded by the mtDNA, was cloned and characterized in 2003 (Antczak et al., 2007). Recently, it was suggested that HsPDF is overexpressed in cancer cells, and inhibitors were developed for further studies (Hu et al., 2020). Our study suggested that HsPDF was downregulated during hypoxia. Furthermore, inhibiting HsPDF both suppressed mtDNA-encoded proteins and mitochondrial OXPHOS in HCC cells. Although the role of HsPDF in regulating translation efficiency is well-reported, further investigation including polysome profiling was needed to confirm ribosome association with these transcripts, which would provide more definitive evidence of impaired translation. In addition, alternative mechanisms, such as protein instability or enhanced degradation pathways, could also contribute to these findings. It’s known that both autophagy and the ubiquitin-proteasome system (UPS) are major pathways for protein degradation. It is possible that these pathways would contribute to the reduced levels of mtDNA-encoded proteins under hypoxia. Future experiments, such as cycloheximide chase and mitochondrial protease inhibition assays will be essential to elucidate the role of protein degradation pathways.

Recently, the role of tRFs in different types of cancer was reported and considered as one of the potential biomarkers for clinical diagnosis as well as prognostic prediction. Lu et al. reported that three tRFs tended to be elevated in the plasma exosomes of patients with colorectal cancer which promoted tumor growth and M2 macrophage polarization (Lu et al., 2022). Mechanistically, tRFs may play important roles in RNA processing, translational regulation and be involved in cellular stress response (Shen et al., 2018). For example, tRF-5 Gln19 interacted via MSC to increase ribosomal and mRNA-binding protein translation (Keam et al., 2017). tRF-3 LeuCAG3, which is highly expressed in HCC, enhanced protein translation efficiency and promoted HCC cell proliferation by binding to the mRNA of ribosomes RPS28 and RPS15 (Kim et al., 2017). Under hypoxic conditions, dicer and angiogenin have been proven to cleave tRNAs into tRFs and tiRNAs. In our study, hypoxia changed tRF expression patterns in HCC cells and that it may cause tRF-3^Thr-CGT^ to suppress HsPDF expression. We hypothesized that hypoxia-induced tRF-3^Thr-CGT^ may be responsible for mtDNA-encoded protein inhibition through targeting HsPDF. Experiments *in vivo* found overexpressed tRF-3^Thr-CGT^ promoted tumor growth significantly. And the observed reduction in the number of tumor nodules when transfected its inhibitor suggested that tRF-3^Thr-CGT^ inhibition may have the potential to suppress tumor cells seeding. These results collectively suggested that tRFs may have roles in mitochondrial protein coordination and may become novel targets for mitochondrial energy remodeling in HCC therapy.

This study has several limitations. For instance, the results were obtained from HCC cell lines and animals while more clinical data is needed to further validate the potential roles of tRF-3^Thr-CGT^ as therapeutic and diagnostic biomarkers. Additionally, nowadays the clinical technology of tRFs is still in its early stages. To develop the *in vivo* research and even further clinical application, delivery method stability and systemic effects should be considered. Recently, studies suggested drug delivery systems such as Synthetic nanoparticles (NPs) and EVs showed advantages in tRFs delivery (Zhang et al., 2025). The engineering modification through genetic editing and chemical modification such as post-transcriptional, Ψ and queuosine (Q) may have potential to improve RNA oligonucleotide stability, while limiting potential off-target effects and delivery efficiency and targeting (Huang et al., 2021; Orellana et al., 2022).

## Data Availability

The data presented in the study are deposited in the NCBI repository, accession number PRJNA1263166.

## Supplementary material

SUPPLEMENTARY FIGURE S1 Hypoxia promoted the HCC cells migration. **(A,B)** Transwell migration assays in SNU-387 and SNU-449 cells, bar = 200 μm. **(C,D)** Scratch Healing Assay at indicated time points in SNU-387 and SNU-449 cells. Data are presented as mean ± SD, n = 3. **p* < 0.05.
