# Supplementary figures and images for: Hypoxia-induced tRF-3Thr-CGT promotes hepatocellular carcinoma progression via mitochondrial energy metabolism remodeling dependent on the mtDNA-translation mechanism
Source: Front Pharmacol. 2025 May 30;16:1549373. doi: 10.3389/fphar.2025.1549373 (PMC12162928; doi:10.3389/fphar.2025.1549373)

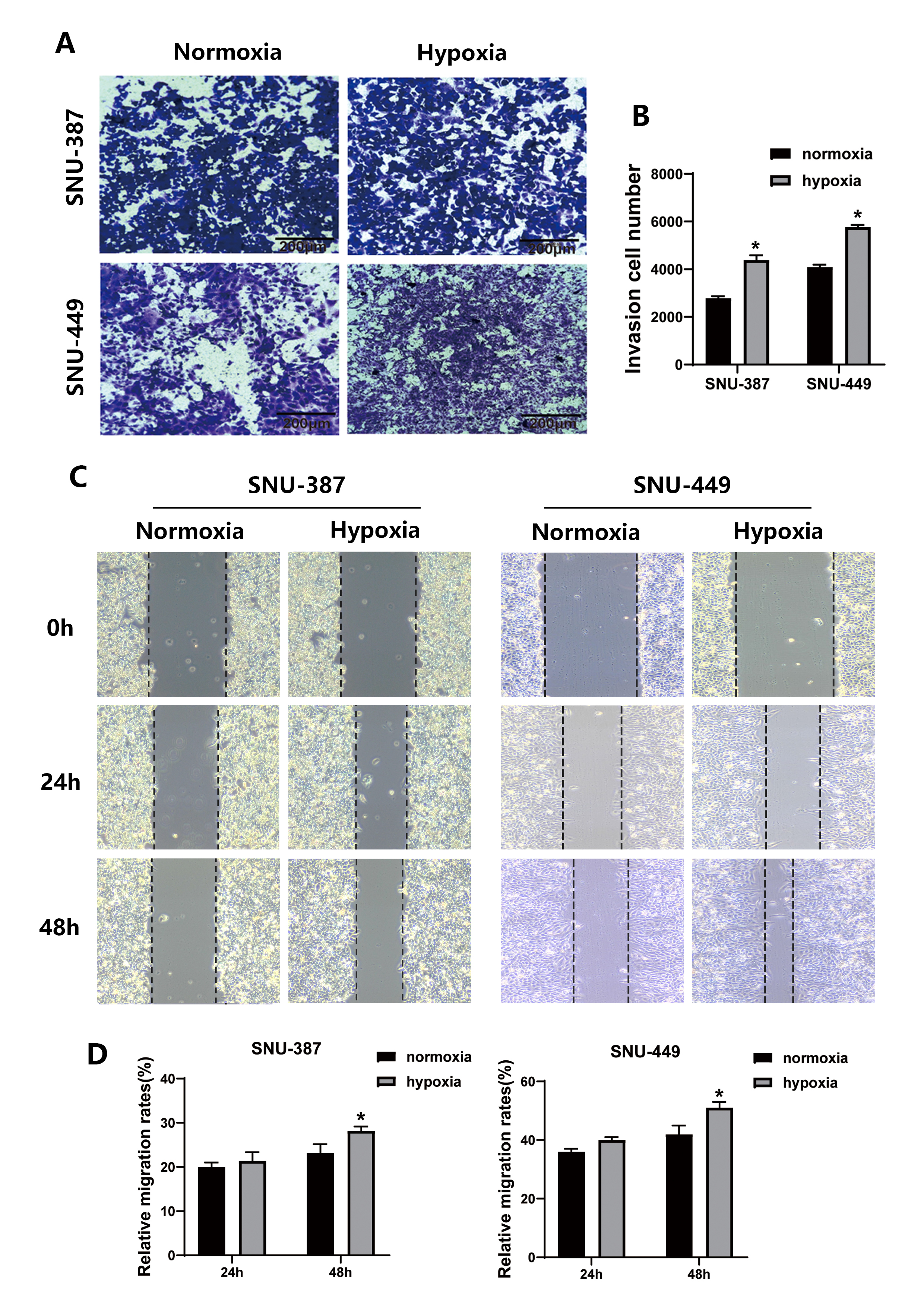

Supplement: Supplementary file 1 [file Image1.jpeg]
